# Supplementary material for: Generating realistic null hypothesis of cancer mutational landscapes using SigProfilerSimulator
Source: BMC Bioinformatics. 2020 Oct 7;21:438. doi: 10.1186/s12859-020-03772-3 (PMC7539472; doi:10.1186/s12859-020-03772-3)

Chromosome 10

Supplementary Figure 1

PD6413a
Breast cancer

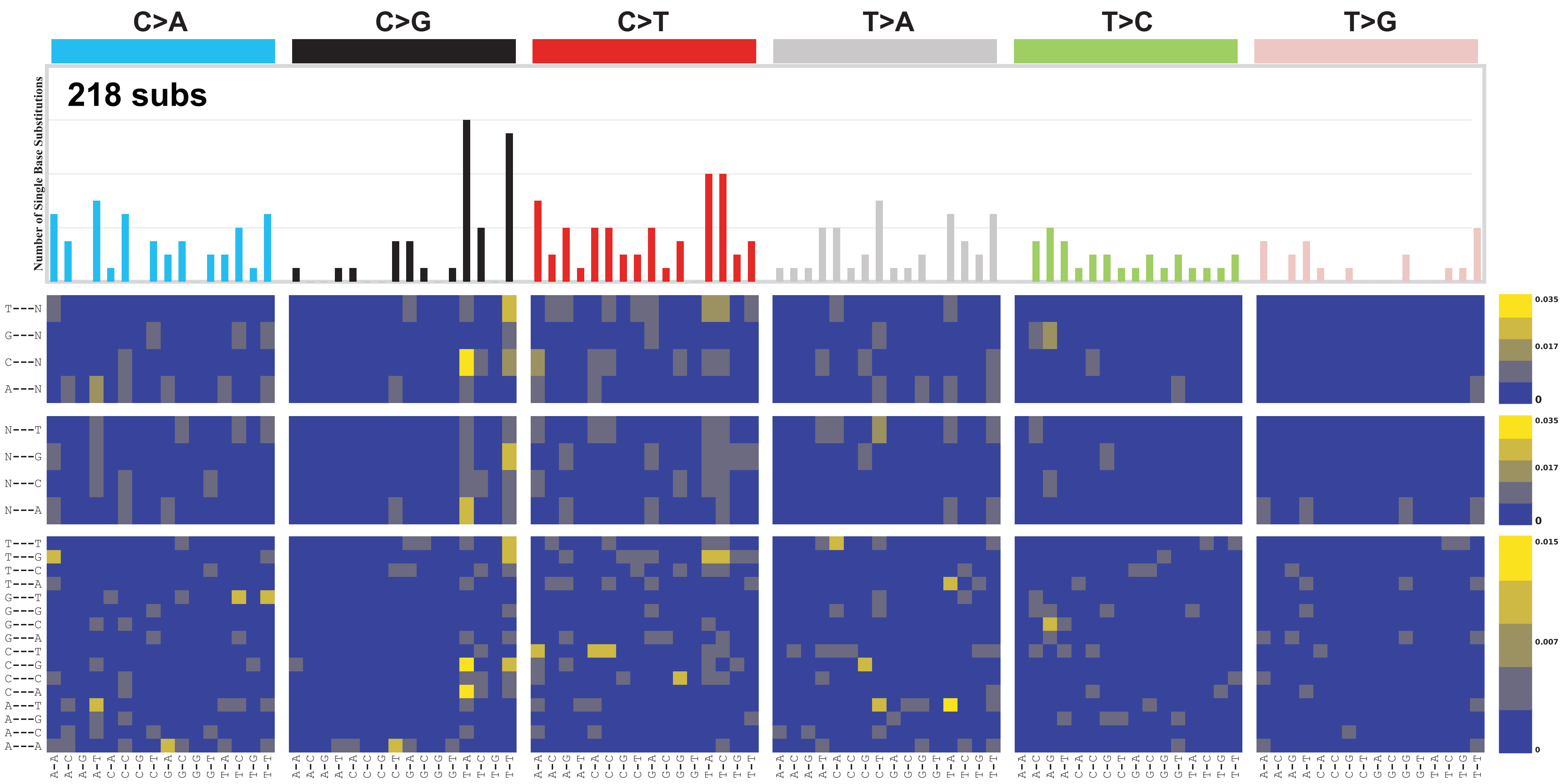

Simulated SBS-1536

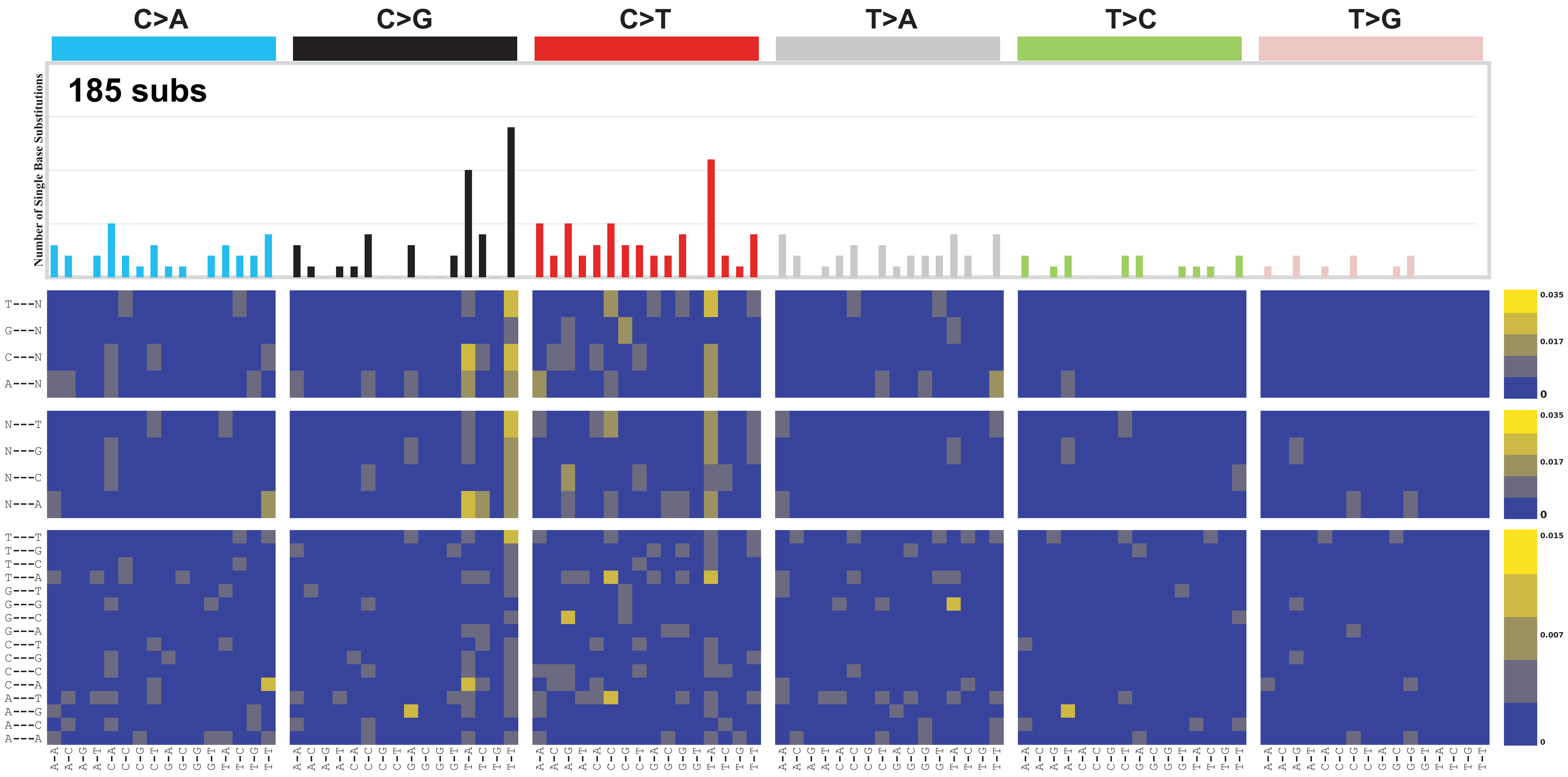

Simulated SBS-1536 per chromosome

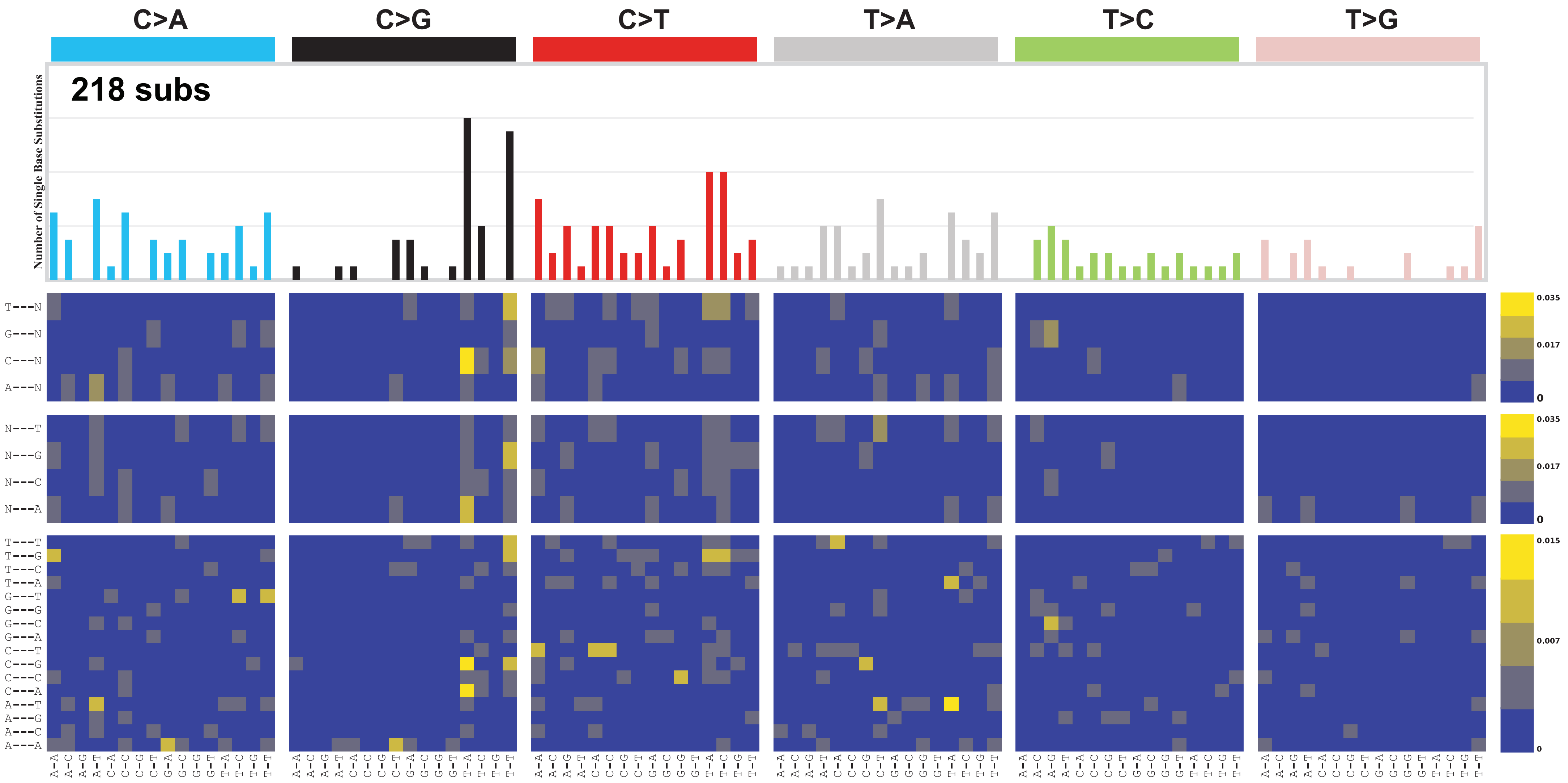

Supplementary Figure 2

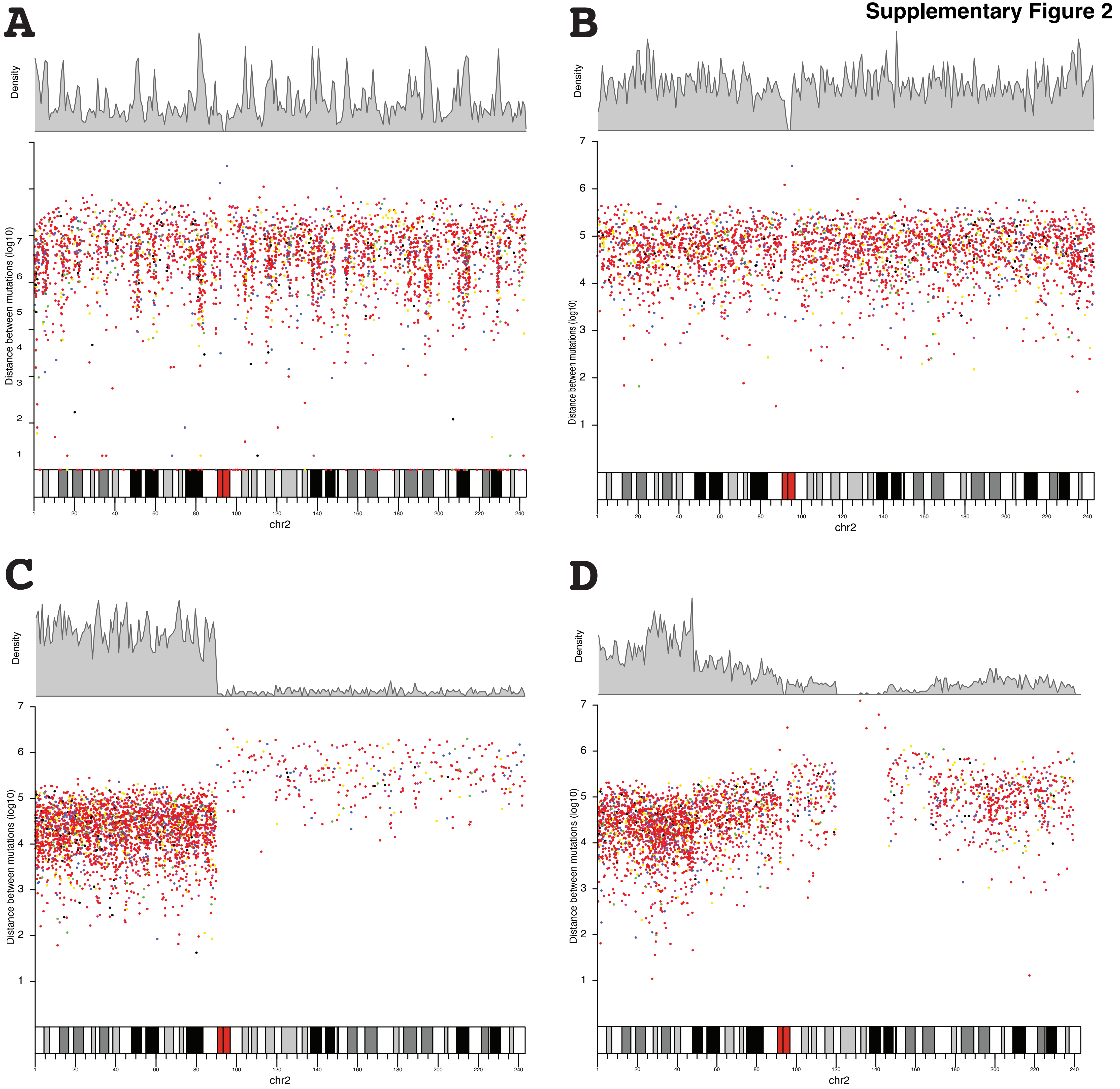

Supplementary Figure 3

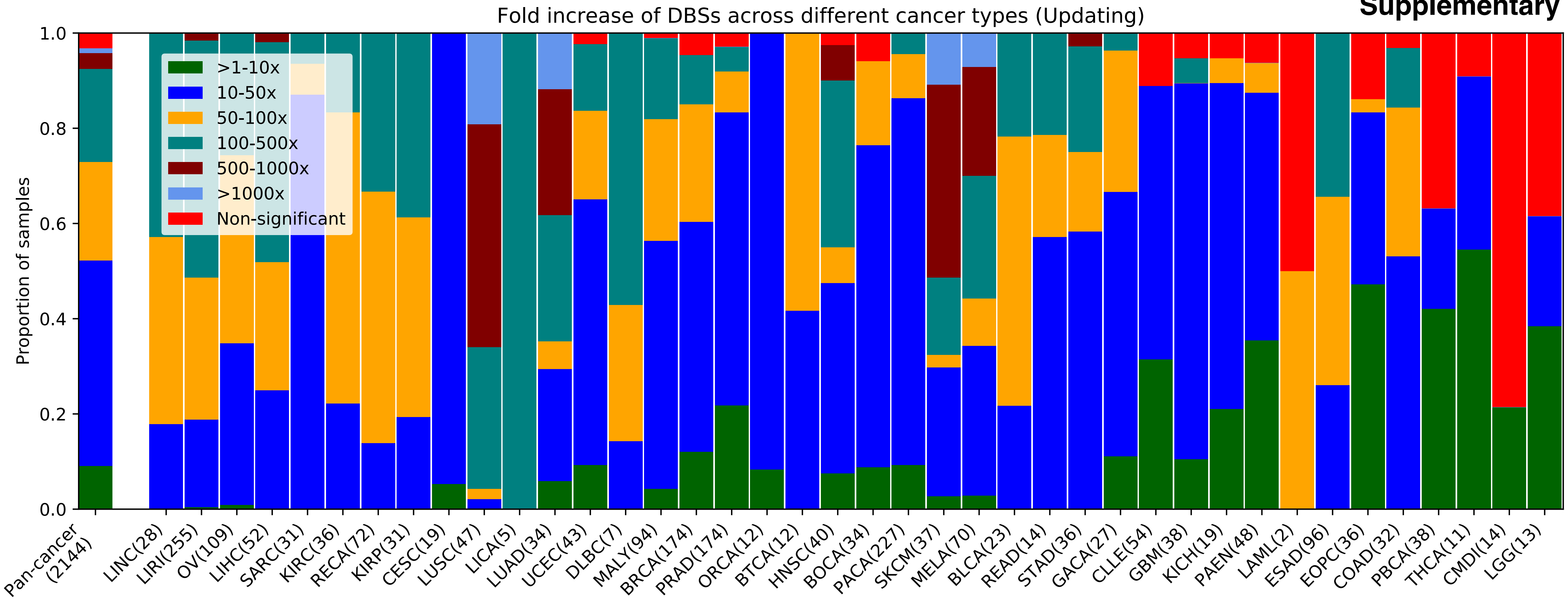

Supplementary Figure 4

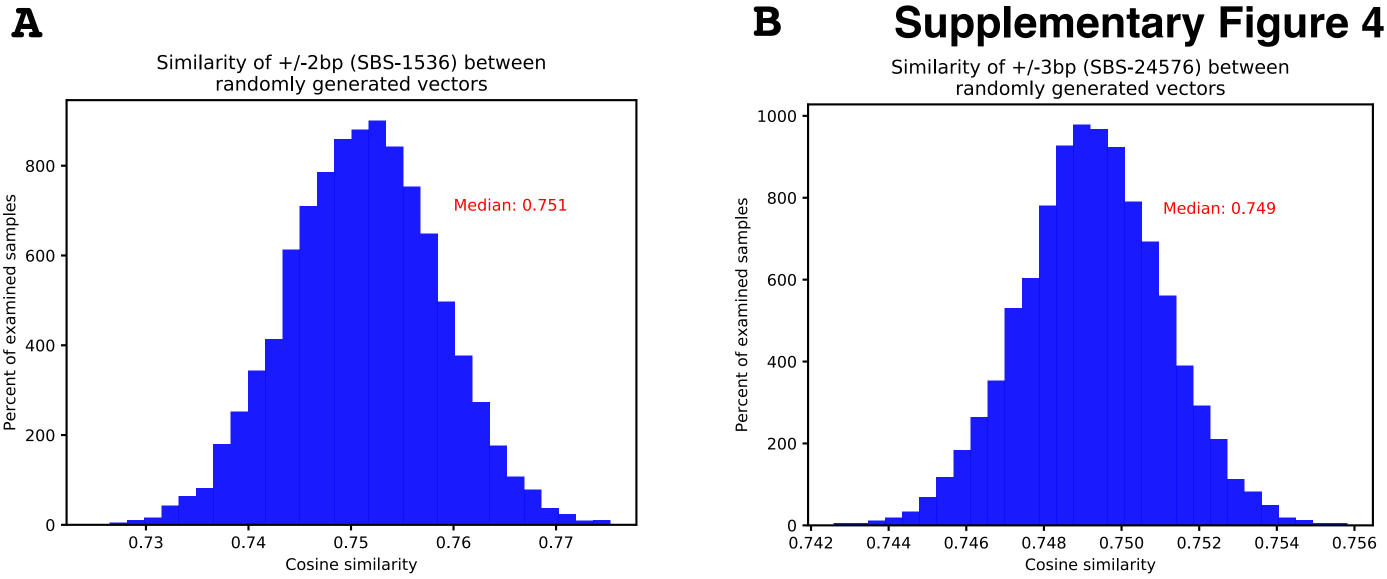

Supplement: Supplementary file 1 — Additional file 1. Figure 1. Example of an additional resolution for simulating mutational patterns supported by SigProfilerSimulator. The example illustrates the resulting patterns when maintaining the mutational burden on each chromosome and when only relying on proportionate allocation based upon the nucleotide context distribution of the reference genome. Comparison is provided for a single breast cancer sample simulated at an SBS-1536 resolution. Figure 2. Simulating cancer genomics data using a probability mask. An example rainfall plot visualization when simulating a single TCGA melanoma sample, TCGA-DA-A-A1I8, with and without a probability mask on chromosome 2. A) Distribution of single base substitutions across chromosome 2 as found in the original sample. B) Distribution of single base substitutions across chromosome 2 when simulating the sample with default parameters. C) Distribution of single base substitutions across chromosome 2 when simulating the sample using a probability mask with 90% probability for mutations on the p arm and a 10% on the q arm. D) Distribution of single base substitutions across chromosome 2 when simulating the sample with a probability mask that varies in weights across the chromosome. All rainfall plots generated using karyoploteR [18]. Y-axes reflect log-scaled distances between adjacent mutations. X-axes reflect positions on chromosome 2 in TCGA-DA-A-A1I8. Each dot reflects a single base substitution colored using the default coloring scheme of karyoploteR. Figure 3. Evaluating the expected rates of DBSs for mutations simulated as dependent events. The fold increase of DBSs observed in the original PCAWG samples and the average number of DBSs observed in our simulations. The mutational pattern of each sample was generated 1000 times considering somatic mutations as dependent events. Figure 4. Evaluating the average similarity of random nonnegative vectors. A) Comparing the cosine similarities amongst 10,000 randomly generat [file 12859_2020_3772_MOESM1_ESM.pdf]
